# Correction: Effects of organic components in cuttlebone on the morphological and mechanical properties of peroxide cross-linked cuttlebone/natural rubber composites

**DOI:** 10.1039/d5ra90125a

**Published:** 2025-11-07

**Authors:** Thitipat Chongcharoenchaikul, Kosuke Miyaji, Preeyanuch Junkong, Sirilux Poompradub, Yuko Ikeda

**Affiliations:** a Graduate School of Science and Technology, Kyoto Institute of Technology Matsugasaki, Sakyo Kyoto 606-8585 Japan; b Department of Chemical Technology, Faculty of Science, Chulalongkorn University Phatumwan Bangkok 10330 Thailand sirilux.p@chula.ac.th; c Center for Rubber Science and Technology, Kyoto Institute of Technology Matsugasaki, Sakyo Kyoto 606-8585 Japan yuko@kit.ac.jp; d Department of Chemistry, Faculty of Science, Mahidol University Ratchathewee Bangkok 10400 Thailand; e Center of Excellence on Petrochemical and Materials Technology, Chulalongkorn University Phatumwan Bangkok 10330 Thailand; f Green Materials for Industrial Application Research Unit, Faculty of Science, Chulalongkorn University Phatumwan Bangkok 10330 Thailand; g Faculty of Molecular Chemistry and Engineering, Kyoto Institute of Technology Matsugasaki, Sakyo Kyoto 606-8585 Japan

## Abstract

Correction for ‘Effects of organic components in cuttlebone on the morphological and mechanical properties of peroxide cross-linked cuttlebone/natural rubber composites’ by Thitipat Chongcharoenchaikul *et al.*, *RSC Adv.*, 2022, **12**, 13557–13565, https://doi.org/10.1039/D2RA01885C.

The ORCiD previously listed for Yuko Ikeda was incorrect. The correct ORCiD is: 0000-0003-2809-8911.

The Royal Society of Chemistry apologises for these errors and any consequent inconvenience to authors and readers.

